# Specific changes in amino acid profiles in monocytes of patients with breast, lung, colorectal and ovarian cancers

**DOI:** 10.3389/fimmu.2023.1332043

**Published:** 2024-01-08

**Authors:** Vitaliy Chagovets, Natalia Starodubtseva, Alisa Tokareva, Anastasia Novoselova, Marina Patysheva, Irina Larionova, Elizaveta Prostakishina, Militsa Rakina, Anna Kazakova, Evgenii Topolnitskiy, Nikolay Shefer, Julia Kzhyshkowska, Vladimir Frankevich, Gennadiy Sukhikh

**Affiliations:** ^1^ National Medical Research Center for Obstetrics Gynecology and Perinatology Named after Academician V.I. Kulakov of the Ministry of Healthcare of Russian Federation, Moscow, Russia; ^2^ Department of Chemical Physics, The Moscow Institute of Physics and Technology, Moscow, Russia; ^3^ Laboratory of Translational Cellular And Molecular Biomedicine, National Research Tomsk State University, Tomsk, Russia; ^4^ Cancer Research Institute, Tomsk National Research Medical Center, Russian Academy of Sciences, Tomsk, Russia; ^5^ Laboratory of Genetic Technologies, Siberian State Medical University, Tomsk, Russia; ^6^ Institute of Transfusion Medicine and Immunology, Mannheim Faculty of Medicine, University of Heidelberg, Heidelberg, Germany; ^7^ German Red Cross Blood Service Baden-Württemberg–Hessen, Mannheim, Germany; ^8^ Laboratory of Translational Medicine, Siberian State Medical University, Tomsk, Russia

**Keywords:** mass spectrometry, metabolomics, oncology, monocytes, tumor-associated macrophages

## Abstract

**Introduction:**

Immunometabolism is essential factor of tumor progression, and tumor-associated macrophages are characterized by substantial changes in their metabolic status. In this study for the first time, we applied targeted amino acid LC-MS/MS analysis to compare amino acid metabolism of circulating monocytes isolated from patients with breast, ovarian, lung, and colorectal cancer.

**Methods:**

Monocyte metabolomics was analyzed by liquid chromatography-mass spectrometry/mass spectrometry (LC-MS/ MS) analysis of amino acid extracts. The targeted analysis of 26 amino acids was conducted by LCMS/MS on an Agilent 6460 triple quadrupole mass spectrometer equipped with an electrospray ionization source and an Agilent 1260 II liquid chromatograph.

**Results:**

Comparison of monocytes of cancer patients with monocytes of healthy control individuals demonstrated that in breast cancer most pronounced changes were identified for tryptophan (AUC = 0.76); for ovarian cancer, aminobutyric acid was significantly elevated (AUC= 1.00); for lung cancer significant changes we indented for citrulline (AUC = 0.70). In order to identify key amino acids that are characteristic for monocytes in specific cancer types, we compared each individual cancer with other 3 types of cancer. We found, that aspartic acid and citrulline are specific for monocytes of patients with colorectal cancer (p<0.001, FC = 1.40 and p=0.003, FC = 1.42 respectively). Citrulline, sarcosine and glutamic acid are ovarian cancer-specific amino acids (p = 0.003, FC = 0.78, p = 0.003, FC = 0.62, p = 0.02, FC = 0.78 respectively). Glutamine, methionine and phenylalanine (p = 0.048, FC = 1.39. p = 0.03, FC = 1.27 and p = 0.02, FC = 1.41) are lung cancer-specific amino acids. Ornithine in monocytes demonstrated strong positive correlation (r = 0.63) with lymph node metastasis incidence in breast cancer patients. Methyl histidine and cysteine in monocytes had strong negative correlation with lymph node metastasis in ovarian cancer patients (r = -0.95 and r = -0.95 respectively). Arginine, citrulline and ornithine have strong negative correlation with tumor size (r = -0.78, citrulline) and lymph node metastasis (r = -0.63 for arginine and r = -0.66 for ornithine).

**Discussion:**

These alterations in monocyte amino acid metabolism can reflect the reaction of systemic innate immunity on the growing tumor. Our data indicate that this metabolic programming is cancer specific and can be inhibiting cancer progression. Cancer-specific differences in citrulline, as molecular link between metabolic pathways and epigenetic programing, provide new option for the development and validation of anti-cancer therapies using inhibitors of enzymes catalyzing citrullination.

## 1 Introduction

Monocytes in the adults represent a dynamic population of precursors of key innate immune cells in tumor microenvironment - tumor associate macrophages (TAMs). Circulating monocytes are differentiated constantly from bone marrow progenitors. Monocytes can spend 1.5 to 4 days in the bloodstream, and can be recruited to the tissues to give rise to macrophages (1–5). Monocyte-derived macrophages in tumor microenvironments can support proliferation of cancer cells, tumor angiogenesis and cancer cells migration and extravasation into blood and lymphatic vessels (6). Different types of monocyte-derived TAMs can have diverse effects on tumor development, growth, angiogenesis, and metastatic progression, as well as suppression of adaptive immune responses (7). In majority of cancers, including breast and lung cancer, both total amount of TAMs as well as their M2 polarization positively correlate with primary tumor growth, metastasis and poor prognosis and decreased survival of patients (8).

Tumor progression is associated with the initiation of a cascade of immune reactions. These pathological processes are linked to changes in both the overall level of peripheral monocytes and their transcriptome and metabolome (9–14). However, majority of the studies were focus on transcriptome.

Monocytes can encounter cancer-related factors in the bloodstream, potentially acquiring programs detrimental to the patient, which are then transferred to tumor tissue, affecting the differentiation of tumor-associated macrophages (TAMs), dendritic cells (DC), and myeloid-derived suppressor cells (MDSC) (12, 15–17). Recent studies utilizing high-throughput methods have shown that transcriptional changes in peripheral blood monocytes can serve as diagnostic, predictive, and prognostic biomarkers in various cancers, including renal, colorectal, breast, cervical, skin, thyroid, hepatocellular, and lung cancers (3, 12, 16–20). The study of the unique features of amino acid metabolism in monocytes across different cancer types deserves special attention. Alterations in amino acid levels can profoundly impact the development of an effective immune response (21–24) and may disrupt the migration, division, and maturation of immune cells. Amino acids play a crucial role in regulating several pathways within immune cells, including mTOR signaling and nitric oxide (NO) production (23, 25). Competition for metabolites and signaling interactions among host immune cells and pathogens can influence disease development (22).

Advancements in analytical techniques, particularly liquid chromatography-mass spectrometry (LC-MS), have led to the emergence of metabolomics (26–28). This field is actively utilized to identify metabolic peculiarities in oncological processes, uncover pathogenesis mechanisms, and identify new therapeutic targets (26, 29). Unlike TAMs, DCs, and MDSCs, which require sophisticated isolation techniques from heterogeneous tumor tissues without compromising highly adherent subpopulations, monocytes are more amenable to examination. Understanding the heterogeneity of monocytes and their roles at various stages of cancer progression is paramount for investigating how cancer cells evade immune surveillance and elude immune cell aggression. Intervening in the pathways and mechanisms involved in monocyte and TAMs reprogramming holds promising potential as a novel therapeutic strategy for personalized cancer immunotherapy (14). We and others have previously identified that monocytes undergo cancer-specific programming that can be detected on the level of phenotype (surface markers) or on the level transcriptome (3, 30, 31). In breast cancer, CD14^low^CD16+HLA-DR+ monocytes were indicative for the good response to neoadjuvant chemotherapy (3). In colon, but not in rectal cancer, overexpression of activator of glycolysis PFKFB3 in monocytes, was correlated with tumor elimination after therapy (30). Metabolism of immune cells is critical for the ability to stimulate or suppress tumor growth. However, despite the monocytes are massively recruited into growing tumor, their metabolic status is almost neglected.

In light of these considerations, our study was designed to analyze the monocyte metabolome in patients with tumors of diverse origins and to examine correlation of cancer-specific amino acid profiles in circulating monocytes with clinical parameters of tumor progression.

## 2 Materials and methods

### 2.1 Study design

The prospective multicenter study included 43 patients with cancer of different localizations (invasive breast carcinoma of no special type, colon and rectal adenocarcinoma, high-grade serous ovarian carcinoma, and lung adenocarcinoma). Patients with breast cancer (n=11) and colorectal cancer (n=16) were treated in the Cancer Research Institute, Tomsk National Research Medical Centre (Tomsk, Russia). Patients with ovarian cancer (n=6) and lung cancer (n=10) were treated in both Cancer Research Institute and Tomsk Regional Oncology Center (Tomsk, Russia). All patients lived in the same geographic area. Patients had no acute pathologies, no infectious disorders, and did not have a history of any other types of cancer in addition to the cancer. Patients did not receive any anticancer treatment prior to sample collection. Inclusion criteria were: primary cancer incident; no history other types of cancers; no anticancer treatment. Exclusion criteria were: acute or chronic infections disorders; pregnancy; alcohol and drug dependence; early-onset of cancer; terminal stage of cancer; cachexia; asthma or autoimmune disorders.

Healthy volunteers (5 women and 5 men) were enrolled in this study as a control group. The inclusion criteria for the healthy cohort were as follows: (a) age from 40 to 77 years, (b) no active medical conditions, (c) not taking immunomodulatory medication (over the counter or prescription) within 30 days of study, and (d) no current or past history of an oncology disease.

Detailed demographic and clinical data of 53 participants included in this study are included in the **Supplementary Table 1** in **Supplementary Material.docx**.

The study received approval from the Local Committee for Medical Ethics and was conducted in accordance with the guidelines of the Declaration of Helsinki and the International Conference on Harmonisation’s Good Clinical Practice Guidelines (ICH GCP). Written informed consent was obtained from all subjects, including the patients/participants, who willingly provided their consent to participate in the study.

### 2.2 Monocyte isolation

Peripheral whole-blood samples were collected from both healthy volunteers (n=10) and patients with cancer of different localizations (n=43). Two millions of monocytes were isolated from 10 ml of blood of a patient. The isolation of monocytes from peripheral blood was performed using density gradients followed by positive magnetic selection with CD14+ MACS beads (#130-050-201, Miltenyi Biotech, Germany) as previously described (30). The isolation quality of monocytes was determined by flow cytometry. Monocyte samples were obtained by positive magnetic separation via СD14 microbeads. Peripheral blood mononuclear cells were the first step in the process. After enrichment on a density gradient, we observed a minimal number of granulocytes in the samples compared to the baseline condition (**Supplementary Figures 1A** and **Supplementary Figure 1B** in Supplementary material.docx). Magnetic separation led to enrichment of monocyte content (**Supplementary Figure 1C** in **Supplementary Material.docx**). Detection by flow cytometry showed a purity of CD14 monocyte isolation of more than 98% (**Supplementary Figures 1D-G** in **Supplementary Material.docx**). After monocyte isolation, the samples were washed twice with DPBS without calcium and magnesium at 300 g for 7 minutes. Subsequently, the cell precipitate obtained was dried under a nitrogen atmosphere and stored at -80°C until further experiments (32).

### 2.3 Amino acid extraction

Monocyte metabolomics was analyzed by liquid chromatography-mass spectrometry/mass spectrometry (LC-MS/MS) analysis of amino acid extracts (32). A 480 µL chloroform-methanol mixture (2:1, v/v) was added at 4°C to the monocyte sample, containing 2*10^6^ cells; sonicated for 10 minutes, following addition of 150 µL of water; centrifuged at 13000 g for 5 minutes at room temperature. The upper aqueous-methanol layer (150 µL) was collected and dried under a stream of nitrogen for 30 minutes at 60°C following addition of 200 µL of 0.1 M hydrochloric acid in butanol for derivatization of amino acids. The solution was mixed for 3 minutes, centrifuged at 13000 g for 15 seconds at room temperature and kept at 60°C for 15 minutes to carry out the derivatization reaction. Afterward, centrifugation was performed at 13000 g for 15 seconds at room temperature. The sample was dried under a stream of nitrogen for 30 minutes at 60°C and reconstituted in 300 µL of acetonitrile/water solution (1:1, v/v). The mixture was stirred for 5 minutes. Centrifugation was carried out at 13000 g for 15 seconds at room temperature for 10 minutes. Finally, 200 µL of the resulting sample was transferred to a vial with an insert for further LC-MS/MS analysis.

### 2.4 Targeted amino acid LC-MS/MS analysis

The targeted analysis of 26 amino acids was conducted by LC-MS/MS on an Agilent 6460 triple quadrupole mass spectrometer (Agilent) equipped with an electrospray ionization source and an Agilent 1260 II liquid chromatograph (Agilent). The system featured a binary high-pressure pump, column thermostat, and an autosampler with 122 vials on an Amino acids-HPLC Column (Jasem) at a temperature of 30°C. The elution mode is provided in **Supplementary Table 2** in **Supplementary Material.docx**, with a flow rate of 0.15 mL/min and a sample volume of 3 µL. Mass spectrometric analysis was performed in positive ion mode at a drying gas temperature and flow rate of 150°C and 10 L/min, respectively. The nebulizer gas pressure was 2.76 bar, the curtain gas temperature and flow rate were 400°C and 10 L/min, respectively, and the voltage was set at 2 kV. MRM transitions for detection are listed in **Supplementary Table 3** in **Supplementary Material.docx**.

No internal standards were used in this study. The reproducibility of analyte extraction was controlled by preparing each sample in triplicate. The difference between preparations did not exceed 15%. Equal amounts of all samples were pooled as a quality control sample to monitor the stability of the LC-MS system and the reproducibility of the analysis. The difference between QC injections did not exceed 10%. The peak areas of amino acids are provided in the **Supplementary_Data.xlxs**, the TIC and EIC of analite were demonstrated in **Supplementary Figure 2** in the **Supplementary Material.docx**.

### 2.5 Statistical analysis

The statistical analysis of experimental data was performed using scripts written in the R language (33) within RStudio (34).

Comparison of amino acid levels between control and cancer groups and comparison between one cancer group and other three was performed by the non-parametric Wilcoxon-Mann-Whitney test. For describing quantitative data, medians (Me) and quartiles Q1 and Q3 were used. The significance level (uncorrected p-value) was set to 0.05. In addition, powers of tests were calculated based on effect size (35) and included in **Supplementary Table 4** in the **Supplementary Material.docx**. Correlations between parameters describing cancer progression (tumor size, number of LNM and hematogenous metastasis) and amino acid levels were assessed using the Spearman rank test, with a significance threshold of p = 0.05.

Logistic regression models for classifying “cancer” vs. “control” were built through stepwise selection of amino acids that exhibited statistically significant differences until there was a reduction in the Akaike Information Criterion (AIC) value. Model quality was evaluated using leave-one-out cross-validation to choose optimal thresholds, sensitivity, and specificity based on maximizing the sum of sensitivity and specificity.

The analysis of the involvement of the cancer specific amino acids in metabolic pathways was conducted using the MetaboAnalyst 5.0 resource (https://www.metaboanalyst.ca/home.xhtml). The pathway impact is calculated as the sum of significance scores of the corresponding metabolites, normalized by the sum of significance scores of all metabolites in each pathway. Enrichment analysis of metabolic pathways was performed using Over Representation Analysis (ORA) employing the hypergeometric test.

## 3 Results

### 3.1 Comparison cancer patients to healthy individuals.

The levels of asparagine, aspartic acid, and tryptophan statistically significantly decreased in patients with breast cancer compared to control group (women) (see **Supplementary Figure 3** and **Supplementary Table 5** in **Supplementary Material.docx**). The levels of citrulline, sarcosine and aminobutyric acid statistically significant decrease in patients with ovarian cancer compared to control group (women) (see **Supplementary Figure 4** and **Supplementary Table 6** in **Supplementary Material.docx**). The level of citrulline statistically significant decrease with the development of lung cancer compared to control (**Supplementary Figure 5** and **Supplementary Table 7** in **Supplementary Material.docx**). In case of colorectal cancer, no statistically significant differences in amino acid profile were identified compared to the healthy donor controls.

Logistic regression models were created for each cancer type classification versus appropriate control group. The formulas are presented below:


$$\text{y}=\frac{1}{1+{\text{e}}^{-9.12+2.61*10^{-3}*{\text{I}}_{\text{Tryptophan}}}}\;(\text{breast cancer vs}.\text{ control},\text{ women}),$$

$$\text{y}=\frac{1}{1+{\text{e}}^{-6.81*10^{2}+6.42*10^{-2}*{\text{I}}_{\text{Aminobutiryc}\;\text{acid}}}}\;(\text{ovarian cancer vs}.\text{ control},\text{ women}),$$

$$\text{y}=\frac{1}{1+{\text{e}}^{-3.15+2.77*10^{-3}*{\text{I}}_{\text{Citrulline}}}}\;(\text{lung cancer vs}.\text{ control}),$$

y is the dependent variable representing the probability of cancer, and I_x_ is the intensity of the amino acid peak X.

**Figure 1:**
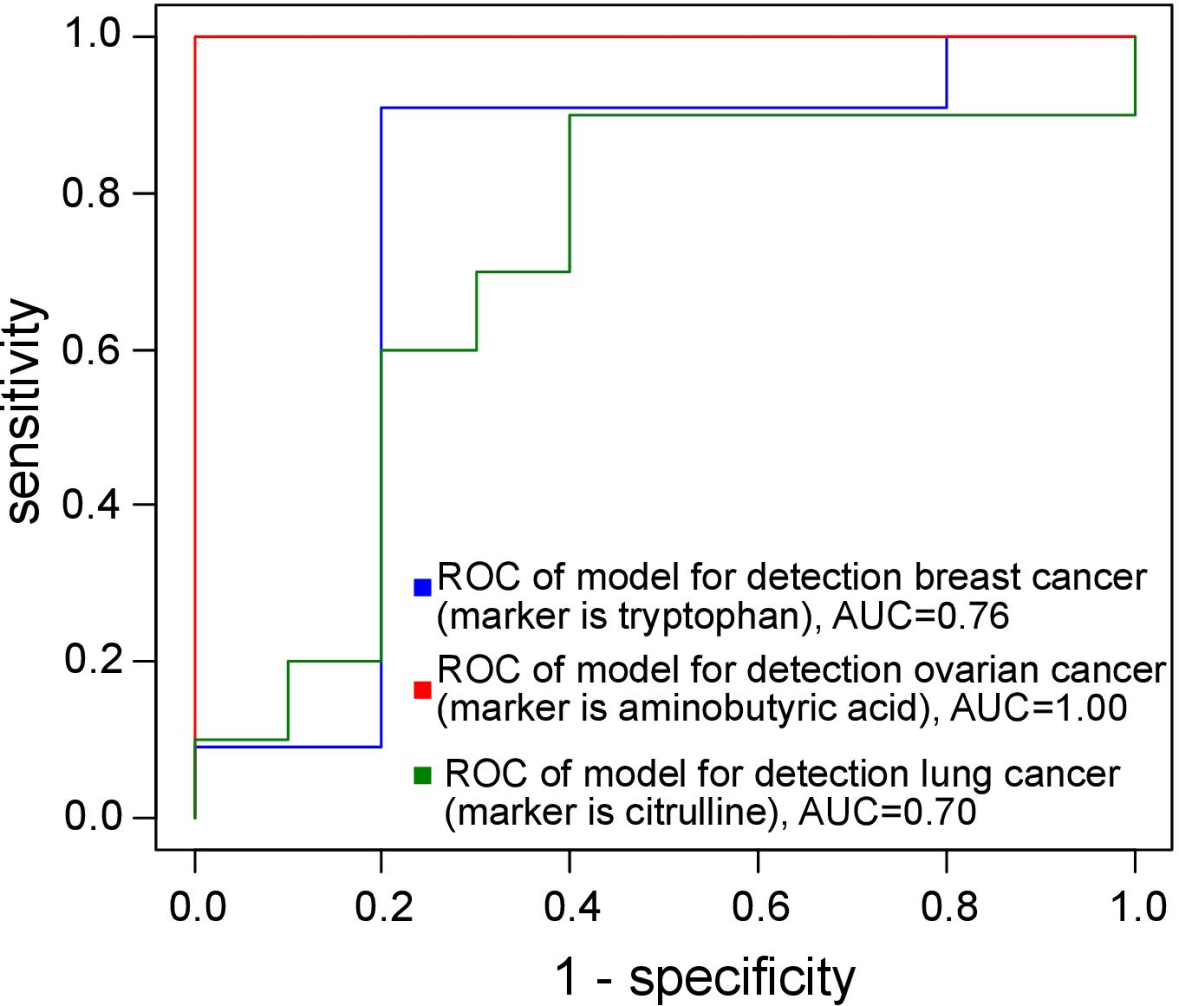
ROC curves of the models for cancer diagnosis.

The diagnostic models exhibited an AUC of at least 0.80 and a sensitivity exceeding 85% (see **Figure 1** and **Table 1**).

**Table 1:** Features of the models suggested for cancer detection based on monocyte amino acid profile.

Cancer	AUC	threshold	sensitivity	specificity
Breast	0.76	0.66	0.91	0.80
Ovarian	1.00	0.001	1	1
Lung	0.70	0.50	0.90	0.70

In breast and ovarian cancers the most pronounced disturbances in amino acid pathways included arginine biosynthesis and alanine, aspartate, and glutamate metabolism (**Supplementary Figure 6**, **Supplementary Tables 8**, **9** in **Supplementary Material** in **Supplementary Material.docx**).

### 3.2 Key differences in amino acid profiles in monocytes of patients identified by direct comparison of monocytes profiles between cancer groups.

**Figure 2:**
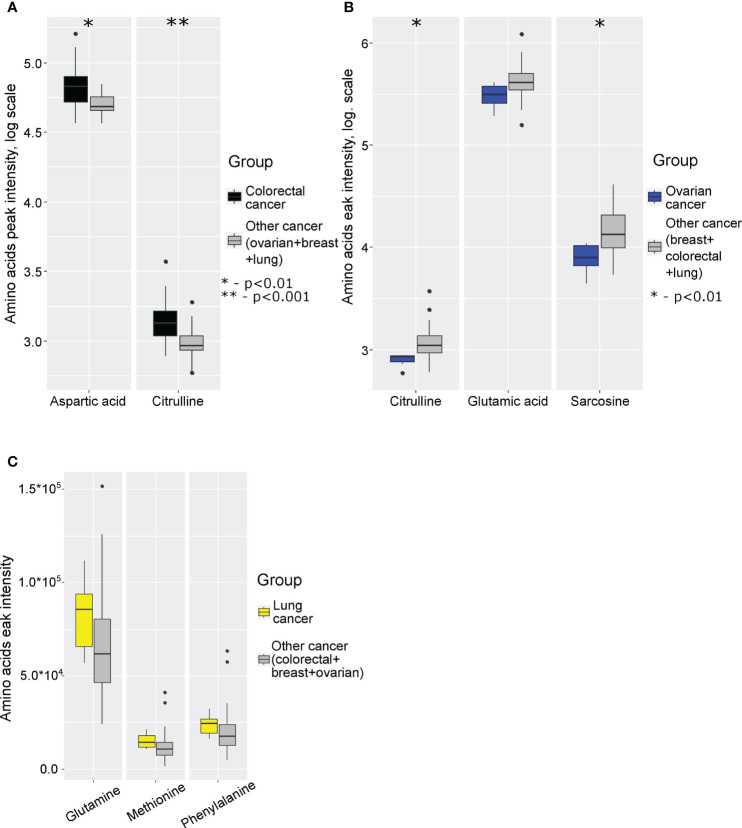
Boxplot of cancer-specific amino acids levels for: **(A)** colorectal cancer; **(B)** ovarian cancer; **(C)** lung cancer. *p (without multiply correction)<0.01, **p (without multiply correction)<0.001. Points represent the outliers.

Next, we analyzed specific changes in amino acid profiles by comparing mass spectrometry data for monocytes isolated from patients with 4 different cancer types. The comparison was performed by analysis of each individual cancer type with the group of three other cancer types. Statistically significant increase of aspartic acid and citrulline levels were detected in case of colorectal cancer (**Figure 2A**; **Supplementary Table 10** in **Supplementary Material.docx**), statistically significant decrease of citrulline, glutamic acid and sarcosine were detected in case of ovarian cancer (**Figure 2B**; **Supplementary Table 11** in **Supplementary Material.docx**), statistically significant increase of glutamine, methionine and phenylalanine levels detected in case of lung cancer (**Figure 2C**; **Supplementary Table 12** in **Supplementary Material.docx**). In case of breast cancer statistically significant differences were not detected.

### 3.3 Correlation of amino acid levels in peripheral blood monocytes correlation with clinical parameters of cancer

**Figure 3:**
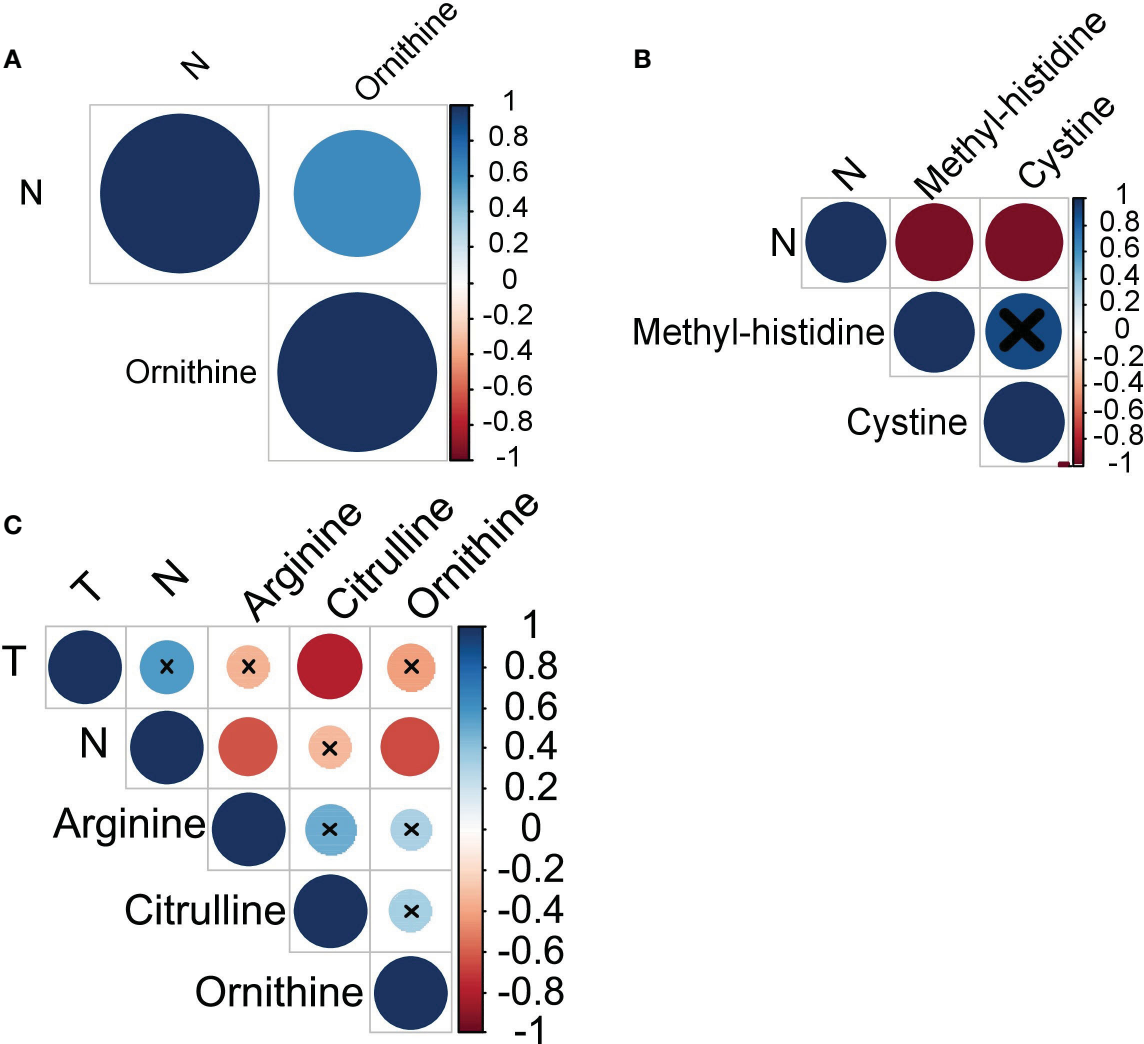
Amino acids exhibiting statistically significant correlations with cancer progression in **(A)** breast cancer, **(B)** ovarian cancer, and **(C)** lung cancer. T, tumor size; N, lymph node metastases.

Next, we have analyzed whether amino acids profiles in monocytes of cancer patients correlate with the clinical parameters of disease progression: tumor size, number of lymph node metastasis and number of hematogenous metastasis. In breast cancer, a strong positive correlation was observed between the levels of ornithine and the number of LNM (r=0.63) (see **Figure 3A**). In ovarian cancer, a very strong negative correlation was found between the number of LNM and the levels of methyl-histidine (r=-0.95) and cystine (r=-0.95) (see **Figure 3B**). Citrulline, arginine and ornithine levels in monocytes of patients with lung cancer negatively correlated with tumor size and LNM (see **Figure 3C**).

## 4 Discussion

In this study, we undertook an effort to unveil the tumor-induced reprogramming of amino acid metabolism in peripheral blood monocytes. We have previously demonstrated that samples suitable for analysis should contain a minimum of 2 × 10^6^ cells (32).

The observed identified differences in monocyte amino acid profiles among patients with different tumors (breast, ovarian, lung, and colorectal cancer) could reflect the long-term impact of tumor cells developing tumor on the metabolism of body in general and, in particular, the immune system (3, 12, 16–21). In cancer patients, the systemic control of monocytes is likely can be achieved through various factors, such as blood cytokines, chemokines, inflammation mediators, exosomes, and lipid and carbohydrate metabolites produced by the tumor (3, 12). Consequently, tumors have the potential to influence both the amount and phenotype of circulating monocytes. Subsequently modified monocyte subpopulations may limit the effectiveness of tumor therapy (36–38). Nevertheless, the role of monocytes as regulators of the tumor’s response to chemotherapeutic agents remains extremely limited (39).

A notable strength of this study lies in the meticulous patient selection and the collection of samples prior to any treatment, effectively mitigating the influence of therapy. It is well-established that various treatment modalities, such as neoadjuvant chemotherapy, surgical procedures, chemotherapy, and radiation therapy, can all exert an impact on blood monocyte levels and their cellular composition (5). Elevated monocyte levels can stem from either increased mobilization from the bone marrow or heightened monopoiesis, both of which have been observed in cancer patients (12). Elevated serum levels of cytokines induced by cancer tumors, combined with low-grade systemic inflammation, may contribute to the distant reprogramming of myelopoiesis (40). Importantly, in several cancer types, patients with higher blood monocyte counts have exhibited a correlation with a less favorable disease prognosis (41–45).

Immune cells face a deprivation of essential amino acids such as tryptophan, cysteine, and arginine (46). In this study, the amino acid profile of peripheral monocytes was dramatically substantially altered in patients with various cancers during the oncogenic process.

In the case of lung and breast cancer, arginine, ornithine and citrulline exhibit strong correlations (r>0.6, p<0.05) with the number of LNM and tumor size. In cancer, arginine metabolism violation is related to immune evasion (promotion of M2-type macrophages), impaired NO synthesis, and increased polyamine production (stimulation of cancer cell proliferation) (47–49). The hypoxic TME increases HIF-1α expression in tumor cells, which in turn stimulate inducible nitric oxide synthase (iNOS) and arginase (ARG1 and ARG2) synthesis (50–52). Enhanced arginase expression is a hallmark of many tumors, such as head and neck, kidney, breast, hepatocellular, and prostate cancers (53–57). Furthermore, anti-inflammatory cytokines released by tumors, such as IL-4, IL-10, and TGF-β1, trigger the activation of ARGs while suppressing iNOS in macrophages (58–60). This process fosters the development of pro-tumoral M2-like macrophages, characterized by heightened ARG activity, reduced iNOS expression, increased proline and polyamine production, and decreased citrulline and NO levels (47, 61, 62). Consistent with these localized pathological mechanisms, a significant reduction in citrulline is observed in peripheral monocytes obtained from patients with lung, and ovarian cancer. Strategies involving the use of ARG1 inhibitors to reinvigorate M1 immunity may, in turn, reactivate tumor-specific Th1 immunity and restore cytotoxic activity (62).

Cancer-induced changes in tryptophan and asparagine in monocytes are of particular interest. Disruptions in tryptophan/asparagine metabolic pathways are associated with tumor processes (21, 22, 26, 63–67). Tryptophan is an essential amino acid, and its availability is a critical factor in the strength and quality of the immune response. The proliferation and activation of human T cells were significantly suppressed in environments lacking tryptophan compared to standard nutrient-rich environments (68, 69). Cancer cells, myeloid-derived suppressor cells (MDSCs), and tumor-associated fibroblasts can reduce tryptophan levels through the enzymatic activity of indoleamine 2,3-dioxygenase (IDO) (21). MDSCs and tumor cells upregulate IDO, inducing an immunosuppressive microenvironment through at least two mechanisms: tryptophan depletion and the accumulation of tryptophan catabolites, such as kynurenine, 3-hydroxyanthranilate, and quinolinate (70, 71). Tryptophan depletion inhibits the proliferation of activated T cells, while catabolites derived from tryptophan act as ligands for aromatic hydrocarbon receptors (72). Kynurenine acts as a suppressor of T-cell immunity, directing the differentiation of naive T cells towards regulatory T cells and suppressing the differentiation of Th17 cells (73).

Intratumoral metabolic status significantly depends on the availability of the nutrients. Tumor reprograms metabolic pathways, promoting metabolic autonomy (74–77). The discovery of oncogenes and tumor suppressors that regulate nutrient uptake and utilization has revealed that nutrients themselves play a key role in cell growth and proliferation (77–80). Significant efforts are being made to develop agents that deplete other amino acids and target central metabolic pathways disrupted in cancer cells, including glycolysis, the tricarboxylic acid cycle, and lipogenesis. Many of these drugs are still in preclinical stages, but some are currently validated in clinical trials (26). The cancer-specific difference in citrulline levels in circulating monocytes are of particular interest since citrulline can link metabolic and epigenetic programs. Citrulline levels can reflect the levels of citrullinated histones. Citrullination of histones is an essential epigenetic mechanism acting both via relaxation of chromatin and as well by interaction with other histone modifications. Enzymes that control citrullination are critical factors of cancer progression, and their expression as well as level of citrullinated proteins in cancer cells and in tumor-associated neutrophils are bad prognostic factors. Inhibitors of these enzymes are under development as cancer therapy drugs (81, 82) However, the role of these enzymes and citrullination of histones in monocytes and TAMs is almost unknown in cancer. Here for the first time we found that levels of citrulline are good prognostic factors for lung cancer. Our data suggest that at least in lung cancer patients citrullination can have a protective role. Thus, the application of inhibitors of the enzymes responsible for the citrullination has to consider their effect also on monocyte-derived TAMs in cancer-specific context.

In conclusion, we found statistically significant cancer-specific changes in the amino acid profile in peripheral blood monocytes in patients who have not received any anti-cancer therapy. Our data suggest that cancer localization has significant impact on the specific changes in the aminoacid profiles in monocytes of patients. The most pronounced changes in amino acid metabolism in monocytes of cancer patients compared to healthy donors were found for breast (decrease in tryptophan and aspartic acid) and ovarian (decrease in citrulline) cancers, and these changes can be indicative for the immunosuppressive programming of monocytes. In contrast, in colorectal cancer monocytes compared to monocytes of patients with other types of cancer, significant increase in aspartic acid and citrulline was identified. This finding provides novel explanation for the anti-tumor activity of TAMs in colorectal cancer, that, in contrast to other types of cancer, retain the ability to inhibit tumor growth. Thus, changes in the aminoacid metabolism in monocytes are involved in the regulation of the balance between pro- and anti-inflammatory activity of macrophages, and can contribute to the pro-tumoral TAM programming during their differentiation from monocytes. Our current study aimed to identify major changes in amino acid metabolism in CD14+ monocytes population that cover over 90% of circulating monocytes, and is composed of both CD14+CD16- and CD14+CD16+ monocytes (83). Due to the limited amount of blood that can be obtained from cancer patients due to ethical restriction, we would be unable to collect sufficient amounts of CD14-CD16+ monocytes to analyse them separately. The other limitations of the study have to be also considered, that include relatively small number of patients for each individual type of cancer, focus of the study on the primary tumors, where majority of patients had no metastasis, absence of analysis of potential gender or age factor contribution, that have to be separately assessed in the large cohort groups in future. However, the statistical reliability of the data suggests the significance of the findings, which can be further validated in larger patient’s cohorts. Correlation of metabolic, transcriptional and epigenetic changes in monocytes will enable identification of the mechanistic links between these major levels of TAMs programming (84). Such new mechanistic links are needed to develop a new generation of TAM-targeted anti-cancer therapy.

## Data availability statement

The datasets presented in this study can be found in online repositories. The names of the repository/repositories and accession number(s) can be found in the article/**Supplementary Material**.

## Ethics statement

The studies involving humans were approved by Ethic Committee of Tomsk National Research Medical Centre, Tomsk, Russia; Ethic Committee of the Tomsk Regional Oncology Center, Tomsk, Russia. The studies were conducted in accordance with the local legislation and institutional requirements. The participants provided their written informed consent to participate in this study.

## Author contributions

VC: Data curation, Investigation, Software, Writing – review & editing. NSt: Conceptualization, Methodology, Writing – original draft, Writing – review & editing. AT: Data curation, Formal analysis, Software, Writing – review & editing. AN: Data curation, Formal analysis, Validation, Writing – review & editing. MP: Resources, Validation, Writing – review & editing. IL: Conceptualization, Validation, Writing – review & editing. EP: Investigation, Resources, Writing – review & editing. MR: Data curation, Visualization, Writing – review & editing. AK: Methodology, Resources, Writing – review & editing. ET: Investigation, Resources, Writing – review & editing. NSh: Conceptualization, Project administration, Writing – review & editing. JK: Conceptualization, Funding acquisition, Writing – review & editing. VF: Conceptualization, Funding acquisition, Writing – review & editing, Writing – original draft. GS: Funding acquisition, Writing – review & editing.
