# Supplementary material for: Specific changes in amino acid profiles in monocytes of patients with breast, lung, colorectal and ovarian cancers
Source: Front Immunol. 2024 Jan 8;14:1332043. doi: 10.3389/fimmu.2023.1332043 (PMC10800720; doi:10.3389/fimmu.2023.1332043)
Supplement: Supplementary file 1 [file DataSheet_1.docx]

Supplementary Material

# Supplementary Data

Supplementary_data: Metabolomics data of the patients.

Supplementary material: Supplementary Figure 1. Evaluation of the purity of CD14+ monocyte after magnetic separation. (A) Peripheral blood leukocyte composition before ficoll density gradient enrichment; (B) Peripheral blood mononuclear cells after isolation by ficoll density gradient; (C) Monocytes after positive CD14 magnetic separation; (D) Scattergram of unstained sample after monocytes isolation; E. Histogram of unstained sample after monocytes isolation; (F) Scattergram of CD14-APC stained sample after monocyte isolation; (G) Histogram of CD14-APC stained sample after monocyte isolation. Supplementary Figure 2. Representative TIC (a) and EIC (b) chromatograms of amino acids from a cell extract. Supplementary Figure 3: Boxplot of levels of amin oacids with statistically significant differences between female control group and breast cancer group. Points represent the outliers; Supplementary Figure 4: Boxplot of levels of amino acids with statistically significant differences between female control group and ovarian cancer group. * - p (without multiply correction) <0.01. Points represent the outliers; Supplementary Figure 5: Boxplot of levels of aminoacids with statistically significant differences between control group and lung cancer group. * - p<0.01; Supplementary Figure 6: Plot of impact on metabolic pathways by lipids and amino acids with statistically significant differences between: (A) control (female) и breast cancer; (B) control (female) and ovarian cancer; Supplementary Table 1. Demographical and clinical data of patients, included in the study; Supplementary Table 2. Eluent gradient for targeted amino acid LC-MS/MS analysis; Supplementary Table 3. MRM-transition of 26 amino acids for mass-spectrometry detection; Supplementary Table 4. Values of power of significantly differences aminoacids; Supplementary Table 5. Top of statistically different amino acids in monocytes of breast cancer patients compared to healthy women; Supplementary Table 6. Top of statistically different amino acids in monocytes of ovarian cancer patients compared to healthy women; Supplementary Table 7. Top of statistically different amino acids in monocytes of lung cancer patients compared to healthy volunteers; Supplementary Table 8. Metabolic pathways of monocytes altered by breast cancer including number of all features in the pathway, probability of zero effect (p), false discovery rate (FDR), and effect on the pathway; Supplementary Table 9. Metabolic pathways of monocytes altered by ovarian cancer including number of all features in the pathway, probability of zero effect (p), false discovery rate (FDR), and effect on the pathway; Supplementary Table 10. Top of statistically different amino acids in monocytes of colorectal cancer patients compared to other patients groups; Supplementary Table 11. Top of statistically different amino acids in monocytes of ovarian cancer patients compared to other patients groups; Supplementary Table 12. Top of statistically different amino acids in monocytes of lung cancer patients compared to other patients groups.

**Supplementary Figure 1.** Evaluation of the purity of CD14+ monocyte after magnetic separation. (A) Peripheral blood leukocyte composition before ficoll density gradient enrichment; (B) Peripheral blood mononuclear cells after isolation by ficoll density gradient; (C) Monocytes after positive CD14 magnetic separation; (D) Scattergram of unstained sample after monocytes isolation; E. Histogram of unstained sample after monocytes isolation; (F) Scattergram of CD14-APC stained sample after monocyte isolation; (G) Histogram of CD14-APC stained sample after monocyte isolation.


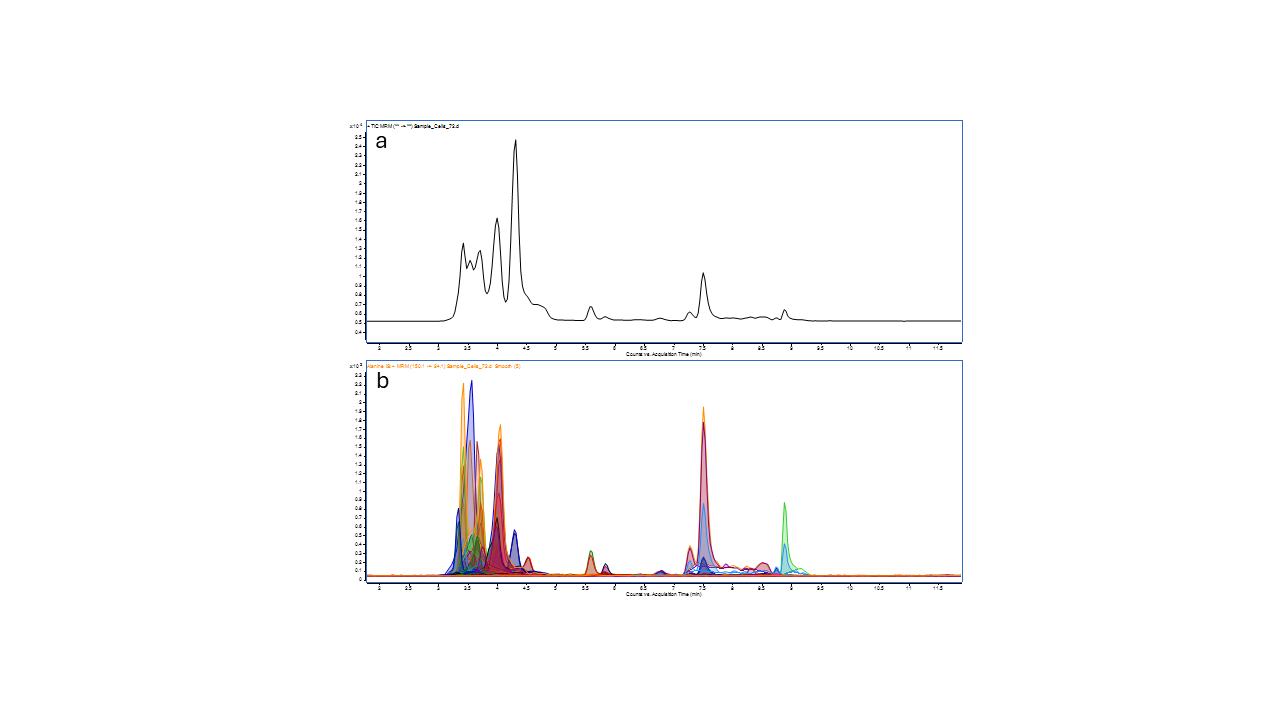


**Supplementary Figure 2.** Representative TIC (a) and EIC (b) chromatograms of amino acids from a cell extract.


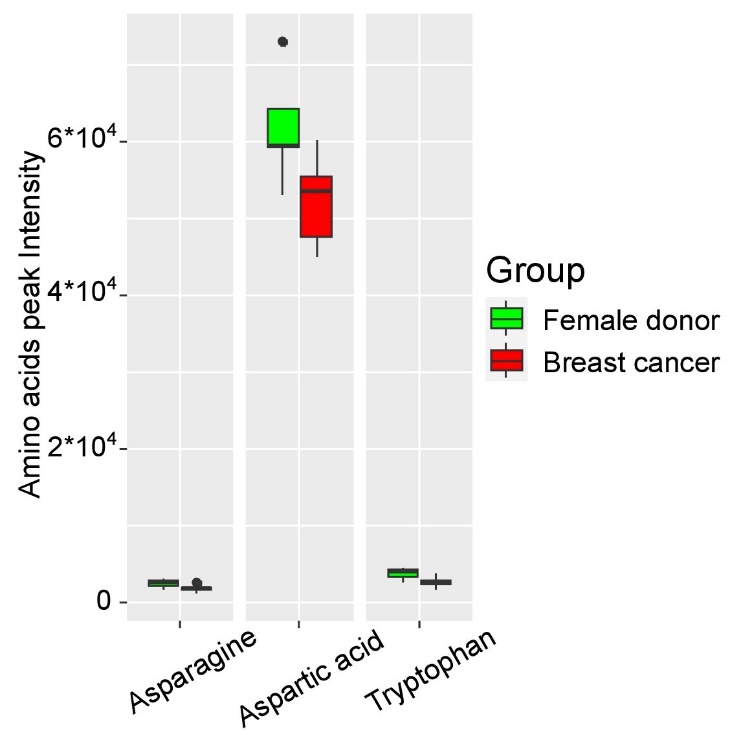


**Supplementary Figure 3.** Boxplot of levels of amin oacids with statistically significant differences between female control group and breast cancer group. Points represent the outliers.


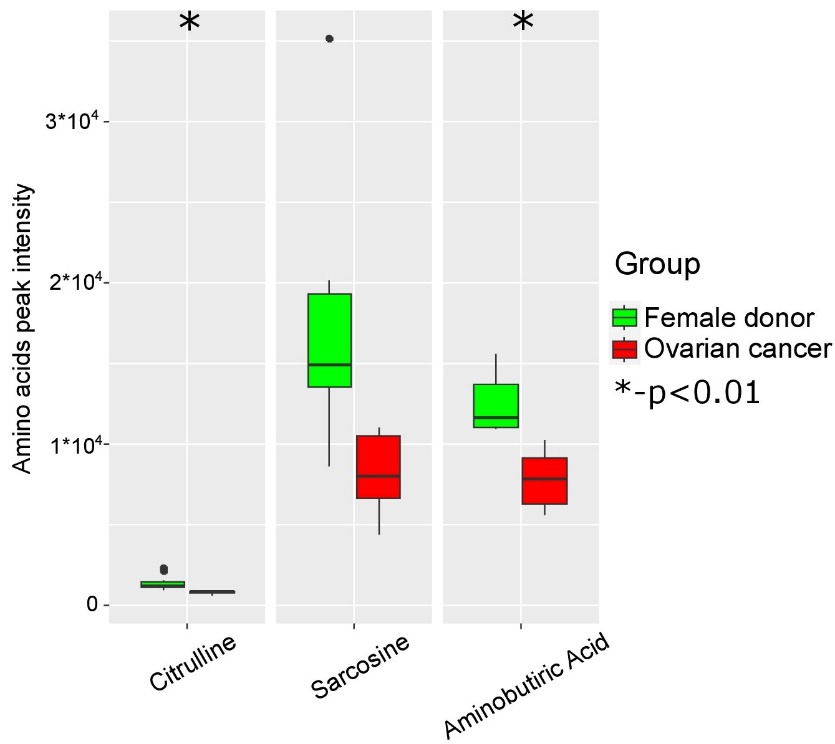


**Supplementary Figure 4:** Boxplot of levels of amino acids with statistically significant differences between female control group and ovarian cancer group. * - p (without multiply correction) <0.01. Points represent the outliers.

**
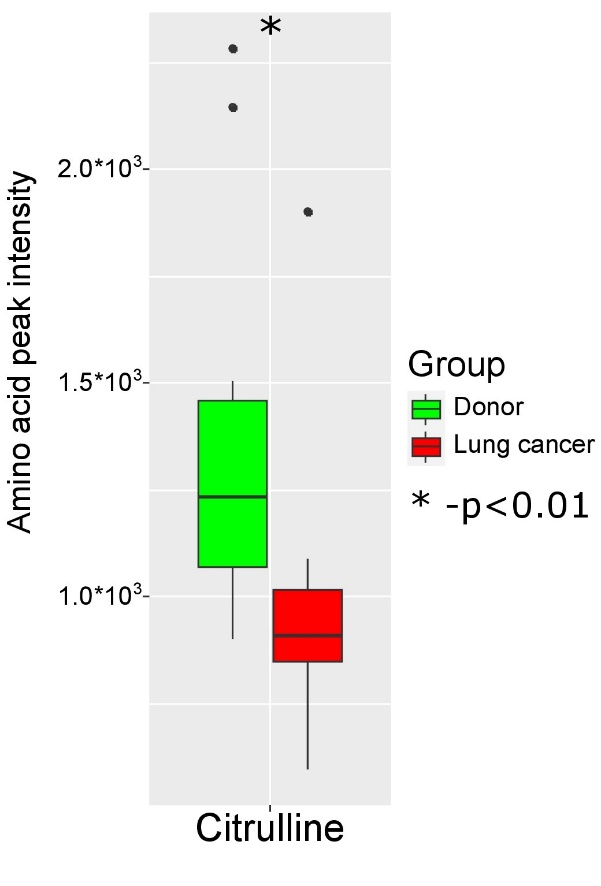
**

**Supplementary Figure 5:** Boxplot of levels of aminoacids with statistically significant differences between control group and lung cancer group. * - p (without multiply correction) <0.01. Points represent the outliers.

**
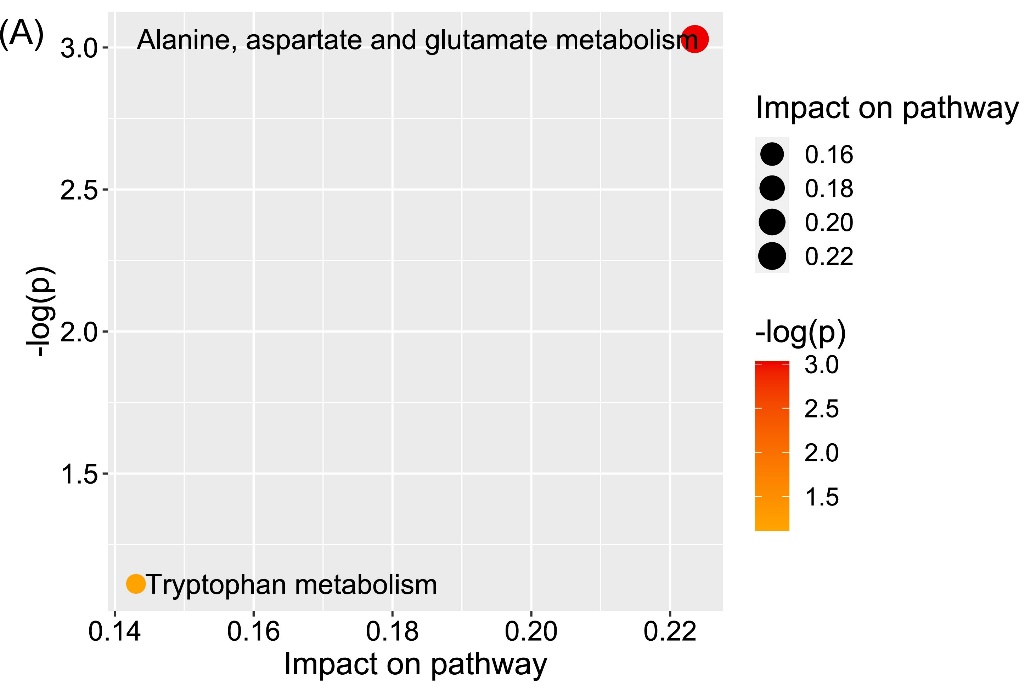
**

**
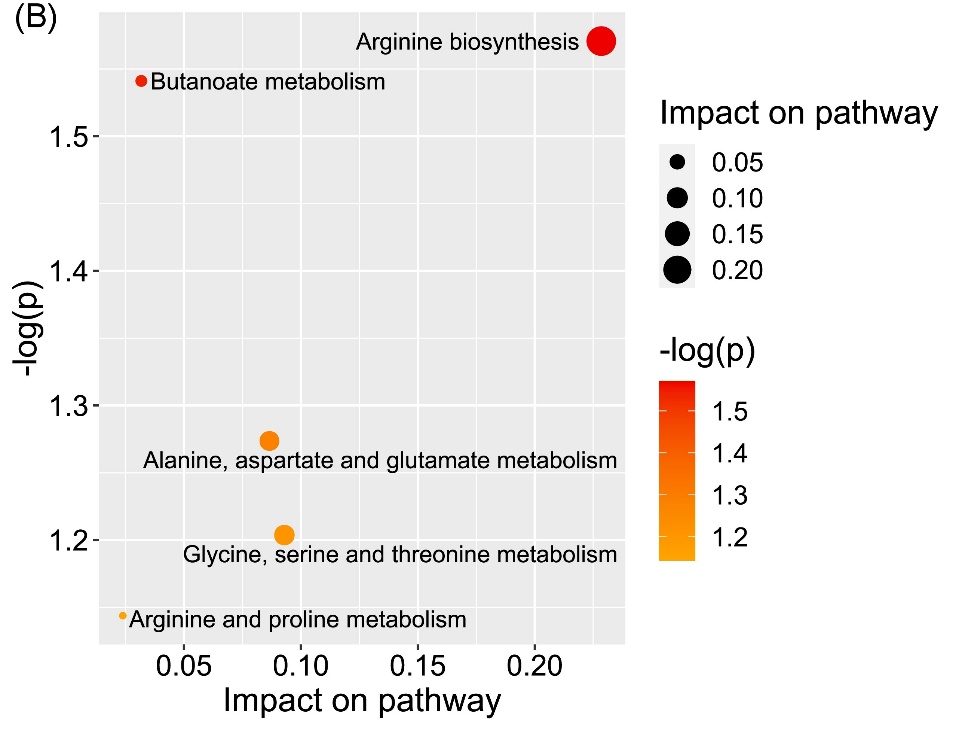
**

**Supplementary Figure 6:** Plot of impact on metabolic pathways by amino acids with statistically significant differences between: (A) control (female) и breast cancer; (B) control (female) and ovarian cancer.

**Supplementary Table 1**. Demographical and clinical data of patients, included in the study.

| Samples | Cancer | age | Sex | Hist. type | T | N | M | Subtype | Cancer type |
| --- | --- | --- | --- | --- | --- | --- | --- | --- | --- |
| bc1 | breast | 51 | Female | ICNT | 2 | 0 | 0 | Her2+ | NA |
| bc11 | breast | 47 | Female | ICNT | 2 | 0 | 0 | Her2+ | NA |
| bc12 | breast | 57 | Female | ICNT | 2 | 2 | 0 | Her2+ | NA |
| bc13 | breast | 61 | Female | ICNT | 1 | 0 | 0 | Her2+ | NA |
| bc2 | breast | 60 | Female | ICNT | 2 | 0 | 0 | Her2+ | NA |
| bc3 | breast | 71 | Female | ICNT | 1 | 0 | 0 | Her2+ | NA |
| bc5 | breast | 55 | Female | ICNT | 2 | 0 | 0 | Her2+ | NA |
| bc6 | breast | 49 | Female | ICNT | 2 | 1 | 0 | Her2+ | NA |
| bc7 | breast | 37 | Female | ICNT | 2 | 1 | 0 | Her2+ | NA |
| bc8 | breast | 64 | Female | ICNT | 1 | 0 | 0 | Her2+ | NA |
| bc9 | breast | 56 | Female | ICNT | 2 | 2 | 0 | Her2+ | NA |
| crc1 | colorectal | 72 | Male | lgauar | 3 | 0 | 0 | NA | rc |
| crc10 | colorectal | 42 | Female | hgasc | 3 | 1 | 0 | NA | cc |
| crc11 | colorectal | 45 | Female | lgarc | 4 | 0 | 0 | NA | rc |
| crc12 | colorectal | 58 | Female | hgarc | 3 | 1 | 0 | NA | rc |
| crc13 | colorectal | 71 | Male | hgasc | 4 | 1 | 1 | NA | cc |
| crc14 | colorectal | 64 | Female | hgasc | 3 | 0 | 0 | NA | cc |
| crc16 | colorectal | 42 | Male | hgarc | 4 | 1 | 1 | NA | rc |
| crc19 | colorectal | 53 | Female | hgauar | 3 | 1 | 0 | NA | rc |
| crc2 | colorectal | 74 | Female | auasc | 3 | 2 | 0 | NA | rc |
| crc3 | colorectal | 67 | Male | hgarj | 4 | 1 | 0 | NA | rc |
| crc4 | colorectal | 70 | Female | lgasc | 3 | 1 | 0 | NA | cc |
| crc5 | colorectal | 42 | Female | cuar | 4 | 1 | 0 | NA | rc |
| crc6 | colorectal | 68 | Male | mdatc | 3 | 0 | 0 | NA | cc |
| crc7 | colorectal | 73 | Male | hgac | 4 | 1 | 0 | NA | cc |
| crc8 | colorectal | 72 | Female | hgasc | 4 | 1 | 0 | NA | cc |
| crc9 | colorectal | 37 | Male | hgauar | 3 | 0 | 0 | NA | rc |
| don1 | control | 44 | Male | NA | NA | NA | NA | NA | NA |
| don10 | control | 59 | Male | NA | NA | NA | NA | NA | NA |
| don2 | control | 55 | Female | NA | NA | NA | NA | NA | NA |
| don3 | control | 74 | Male | NA | NA | NA | NA | NA | NA |
| don4 | control | 72 | Female | NA | NA | NA | NA | NA | NA |
| don5 | control | 54 | Male | NA | NA | NA | NA | NA | NA |
| don6 | control | 51 | Female | NA | NA | NA | NA | NA | NA |
| don7 | control | 65 | Male | NA | NA | NA | NA | NA | NA |
| don8 | control | 40 | Female | NA | NA | NA | NA | NA | NA |
| don9 | control | 54 | Female | NA | NA | NA | NA | NA | NA |
| lc1 | lung | 68 | Male | mdscc | 2 | 0 | 0 | NA | NA |
| lc10 | lung | 75 | Male | pdscc | 3 | 0 | 0 | NA | NA |
| lc2 | lung | 59 | Male | nscc | 2 | 0 | 0 | NA | NA |
| lc3 | lung | 68 | Male | mdscc | 4 | 2 | 0 | NA | NA |
| lc4 | lung | 66 | Male | nscc | 2 | 0 | 0 | NA | NA |
| lc5 | lung | 47 | Male | nscc | 3 | 2 | 0 | NA | NA |
| lc6 | lung | 63 | Male | nscc | 2 | 0 | 0 | NA | NA |
| lc7 | lung | 62 | Male | nscc | 3 | 1 | 0 | NA | NA |
| lc8 | lung | 73 | Male | nscc | 3 | 0 | 0 | NA | NA |
| lc9 | lung | 74 | Female | nscc | 2 | 1 | 0 | NA | NA |
| oc1 | ovarian | 66 | Female | hgsoc | 3 | 2 | 0 | NA | NA |
| oc10 | ovarian | 40 | Female | hgsoc | 3 | 0 | 0 | NA | NA |
| oc5 | ovarian | 64 | Female | hgsoc | 3 | 2 | 1 | NA | NA |
| oc7 | ovarian | 71 | Female | hgsoc | 3 | 0 | 0 | NA | NA |
| oc8 | ovarian | 71 | Female | hgsoc | 3 | 0 | 0 | NA | NA |
| oc9 | ovarian | 63 | Female | hgsoc | 3 | 1 | 0 | NA | NA |

**Supplementary Table 2.** Eluent gradient for chromatography separation of aminoacids sample. A - 10 мМ ammonium acetate solution in water; B – acetonitrile.

| Time, min | A% | B% |
| --- | --- | --- |
| 0.00 | 95 | 5 |
| 0.10 | 95 | 5 |
| 0.20 | 75 | 25 |
| 5.00 | 70 | 30 |
| 15.00 | 45 | 55 |
| 20.00 | 30 | 70 |
| 20.50 | 5 | 95 |
| 25.00 | 5 | 95 |
| 25.50 | 95 | 5 |
| 40.00 | 95 | 5 |

**Supplementary Table 3.** MRM-transition of amino acids mass-spectrometry detection.

| # | **Compound Group** | **Precur-sor Ion** | **Pro-duct Ion** | **Dwell** | **Frag-mentor** | **Collision Energy** | **Cell Accelerator Voltage** | **Polarity** |
| --- | --- | --- | --- | --- | --- | --- | --- | --- |
| 1 | 2-Aminobutiric_Acid | 160.1 | 143.1 | 2 | 83 | 4 | 4 | Positive |
|  |  | 160.1 | 87.1 | 2 | 83 | 12 | 4 | Positive |
|  |  | 160.1 | 86.2 | 2 | 83 | 8 | 4 | Positive |
|  |  | 160.1 | 58.2 | 2 | 83 | 12 | 4 | Positive |
|  |  | 160.1 | 45.2 | 2 | 83 | 32 | 4 | Positive |
|  |  | 160.1 | 41.2 | 2 | 83 | 28 | 4 | Positive |
|  |  | 160.1 | 30.2 | 2 | 83 | 12 | 4 | Positive |
| 2 | Methyl-Histidine | 226.16 | 109.1 | 2 | 122 | 20 | 4 | Positive |
|  |  | 226.16 | 42.2 | 2 | 122 | 76 | 4 | Positive |
|  |  | 226.16 | 68.2 | 2 | 122 | 56 | 4 | Positive |
|  |  | 226.16 | 96.1 | 2 | 122 | 24 | 4 | Positive |
| 3 | 5-OH-Lysine | 219.2 | 128 | 2 | 83 | 12 | 4 | Positive |
|  |  | 219.2 | 82.2 | 2 | 83 | 20 | 4 | Positive |
| 4 | Alanine | 146.1 | 39.2 | 2 | 83 | 20 | 4 | Positive |
|  |  | 146.1 | 44.2 | 2 | 83 | 12 | 4 | Positive |
|  |  | 146.1 | 57.2 | 2 | 83 | 12 | 4 | Positive |
|  |  | 146.1 | 90.1 | 2 | 83 | 4 | 4 | Positive |
| 5 | Arginine | 231.18 | 172.1 | 2 | 83 | 12 | 4 | Positive |
|  |  | 231.18 | 70.2 | 2 | 83 | 28 | 4 | Positive |
| 6 | Asparagine | 189.1 | 144.1 | 2 | 83 | 8 | 4 | Positive |
|  |  | 189.1 | 74.2 | 2 | 83 | 16 | 4 | Positive |
| 7 | Aspartic_Acid | 246.2 | 144.1 | 2 | 83 | 8 | 4 | Positive |
|  |  | 246.2 | 88.1 | 2 | 83 | 16 | 4 | Positive |
| 8 | Citrulline | 232.2 | 215.1 | 2 | 83 | 4 | 4 | Positive |
|  |  | 232.2 | 70.2 | 2 | 83 | 28 | 4 | Positive |
| 9 | Cystine | 353.16 | 130.1 | 2 | 122 | 20 | 4 | Positive |
|  |  | 353.16 | 74.1 | 2 | 122 | 36 | 4 | Positive |
| 10 | Glutamic_Acid | 260.19 | 186.1 | 2 | 83 | 8 | 4 | Positive |
|  |  | 260.19 | 84.1 | 2 | 83 | 24 | 4 | Positive |
| 11 | Glutamine | 203.14 | 186.1 | 2 | 83 | 4 | 4 | Positive |
|  |  | 203.14 | 41.2 | 2 | 83 | 36 | 4 | Positive |
|  |  | 203.14 | 56.2 | 2 | 83 | 40 | 4 | Positive |
|  |  | 203.14 | 84.1 | 2 | 83 | 20 | 4 | Positive |
| 12 | Glycine | 132.1 | 76.2 | 2 | 44 | 4 | 4 | Positive |
|  |  | 132.1 | 57.2 | 2 | 44 | 12 | 4 | Positive |
| 13 | Histidine | 212.14 | 110.1 | 2 | 83 | 12 | 4 | Positive |
|  |  | 212.14 | 83.2 | 2 | 83 | 32 | 4 | Positive |
| 14 | Leucine | 188.17 | 30.3 | 2 | 83 | 20 | 4 | Positive |
|  |  | 188.17 | 44.3 | 2 | 83 | 32 | 4 | Positive |
|  |  | 188.17 | 86.2 | 2 | 83 | 8 | 4 | Positive |
| 15 | Lysine | 203.2 | 130.1 | 2 | 83 | 8 | 4 | Positive |
|  |  | 203.2 | 186.2 | 2 | 83 | 4 | 4 | Positive |
|  |  | 203.2 | 56.2 | 2 | 83 | 48 | 4 | Positive |
|  |  | 203.2 | 84.2 | 2 | 83 | 20 | 4 | Positive |
| 16 | Methionine | 206.12 | 104.1 | 2 | 83 | 8 | 4 | Positive |
|  |  | 206.12 | 56.2 | 2 | 83 | 20 | 4 | Positive |
| 17 | Ornithine | 189.2 | 172.2 | 2 | 83 | 8 | 4 | Positive |
|  |  | 189.2 | 70.2 | 2 | 83 | 20 | 4 | Positive |
| 18 | Phenilalnine | 222.1 | 103.1 | 2 | 83 | 40 | 4 | Positive |
|  |  | 222.1 | 77.2 | 2 | 83 | 56 | 4 | Positive |
| 19 | Proline | 172.14 | 116.1 | 2 | 83 | 12 | 4 | Positive |
|  |  | 172.14 | 70.2 | 2 | 83 | 16 | 4 | Positive |
| 20 | Sarcosine | 146.1 | 44.3 | 2 | 83 | 12 | 4 | Positive |
|  |  | 146.1 | 57.2 | 2 | 83 | 16 | 4 | Positive |
|  |  | 146.1 | 72.2 | 2 | 83 | 8 | 4 | Positive |
|  |  | 146.1 | 90.1 | 2 | 83 | 8 | 4 | Positive |
| 21 | Serine | 162.12 | 106.1 | 2 | 83 | 8 | 4 | Positive |
|  |  | 162.12 | 60.2 | 2 | 83 | 12 | 4 | Positive |
| 22 | Threonine | 176.13 | 158.1 | 2 | 83 | 4 | 4 | Positive |
|  |  | 176.13 | 74.2 | 2 | 83 | 12 | 4 | Positive |
| 23 | Trans-4-OH-Proline | 188.1 | 86.2 | 2 | 83 | 12 | 4 | Positive |
|  |  | 188.1 | 68.2 | 2 | 83 | 32 | 4 | Positive |
| 24 | Tryptophan | 261.16 | 244.1 | 2 | 83 | 4 | 4 | Positive |
|  |  | 261.16 | 117.1 | 2 | 83 | 48 | 4 | Positive |
| 25 | Tyrosine | 238.1 | 136.1 | 2 | 83 | 12 | 4 | Positive |
|  |  | 238.1 | 91.1 | 2 | 83 | 40 | 4 | Positive |
| 26 | Valine | 174.15 | 72.2 | 2 | 83 | 12 | 4 | Positive |
|  |  | 174.15 | 55.2 | 2 | 83 | 38 | 4 | Positive |

**Supplementary Table 4.** Power values of monocyte amino acid parameters significantly differed between the study groups.

| Task | Amino acid | Power |
| --- | --- | --- |
| ovarian cancer vs female donor | Citrulline | 0.79 |
|  | Sarcosine | 0.51 |
|  | Aminobutiric acid | 0.98 |
| breast cancer vs donor | Asparagine | 0.77 |
|  | Tryptophan | 0.89 |
|  | Aspartic acid | 0.84 |
| lung cancer vs donor | Citrulline | 0.53 |

**Supplementary Table 5.** Top of statistically different amino acids in monocytes of breast cancer patients compared to healthy women.

| Amino acid | Control (female) | Breast cancer | P | FC |
| --- | --- | --- | --- | --- |
| Asparagine | 2.67*10^3^(2.26*10^3^;2.87*10^3^) | 1.83*10^3^(1.57*10^3^;1.94*10^3^) | 0.04 | 0.68 |
| Tryptophan | 4.04*10^3^(3.43*10^3^;4.2*10^3^) | 2.66*10^3^(2.35*10^3^;2.96*10^3^) | 0.01 | 0.66 |
| Aspartic acid | 5.96*10^4^(5.92*10^4^;6.43*10^4^) | 5.34*10^4^(4.75*10^4^;5.54*10^4^) | 0.04 | 0.90 |

**Supplementary Table 6.** Top of statistically different amino acids in monocytes of ovarian cancer patients compared to healthy women.

| Amino acids | Control (female) | Ovarian cancer | P | FC |
| --- | --- | --- | --- | --- |
| Citrulline | 1.19*10^3^(1.12*10^3^;2.15*10^3^) | 0.86*10^3^(0.76*10^3^;0.87*10^3^) | 0.004 | 0.72 |
| Sarcosine | 13.5*10^3^(12.6*10^3^;14.5*10^3^) | 8.05*10^3^(6.64*10^3^;10.5*10^3^) | 0.03 | 0.60 |
| Aminobutiric acid | 11.4*10^3^(11.2*10^3^;11.9*10^3^) | 7.82*10^3^(6.25*10^3^;9.13*10^3^) | 0.004 | 0.69 |

**Supplementary Table 7.** Top of statistically different amino acids in monocytes of lung cancer patients compared to healthy volunteers.

| Amino acid | Control | Lung cancer | P | FC |
| --- | --- | --- | --- | --- |
| Citrulline | 1.24*10^3^ (1.07*10^3^;1.46*10^3^) | 0.91*10^3^ (0.85*10^3^;1.02*10^3^) | 0.001 | 0.74 |

**Supplementary Table 8.** Metabolic pathways of monocytes altered by breast cancer including number of all features in the pathway, probability of zero effect (p), false discovery rate (FDR), and effect on the pathway.

| Pathways | Total | Hits | P | FDR | Impact |
| --- | --- | --- | --- | --- | --- |
| Alanine, aspartate and glutamate metabolism | 28 | 2 | <0.001 | 0.04 | 0.22 |
| Tryptophan metabolism | 41 | 1 | 0.08 | 0.81 | 0.14 |

**Supplementary Table 9.** Metabolic pathways of monocytes altered by ovarian cancer including number of all features in the pathway, probability of zero effect (p), false discovery rate (FDR), and effect on the pathway.

| Pathways | Total | Hits | P | FDR | Impact |
| --- | --- | --- | --- | --- | --- |
| Arginine biosynthesis | 14 | 1 | 0.03 | 1.00 | 0.23 |
| Butanoate metabolism | 15 | 1 | 0.08 | 1.00 | 0.03 |
| Alanine, aspartate and glutamate metabolism | 28 | 1 | 0.05 | 1.00 | 0.09 |
| Glycine, serine and threonine metabolism | 33 | 1 | 0.06 | 1.00 | 0.09 |
| Arginine and proline metabolism | 38 | 1 | 0.07 | 1.00 | 0.02 |

**Supplementary Table 10.** Top of statistically different amino acids in monocytes of colorectal cancer patients compared to other patients groups.

| Amino acids | Other Localization | Colorectal | P | FC |
| --- | --- | --- | --- | --- |
| Citrulline | 0.93*10^3^(0.85*10^3^;1.1*10^3^) | 1.3*10^3^(1.1*10^3^;1.6.0*10^3^) | <0.001 | 1.40 |
| Aspartic acid | 4.8*10^4^(4.5*10^4^;5.7*10^4^) | 6.8*10^4^(5.2*10^4^;7.9*10^4^) | 0.003 | 1.42 |

**Supplementary Table 11.** Top of statistically different amino acids in monocytes of ovarian cancer patients compared to other patients groups.

| Amino acid | Other localization | Ovarian cancer | P | FC |
| --- | --- | --- | --- | --- |
| Citrulline | 1.1*10^3^(9.3*10^2^;1.4*10^3^) | 8.6*10^2^(7.6*10^2^;8.7*10^2^) | 0.003 | 0.78 |
| Sarcosine | 1.3*10^4^(9.9*10^3^;2.1*10^4^) | 8*10^3^(6.6*10^3^;1*10^4^) | 0.003 | 0.62 |
| Glutamic acid | 4.1*10^5^(3.5*10^5^;5*10^5^) | 3.2*10^5^(2.6*10^5^;3.8*10^5^) | 0.02 | 0.78 |

**Supplementary Table 12.** Top of statistically different amino acids in monocytes of lung cancer patients compared to other patients groups.

| Amino acid | Other localization | Lung | P | FC |
| --- | --- | --- | --- | --- |
| Glutamine | 6.2*10^4^(4.6*10^4^;8.0*10^4^) | 8.6*10^4^(6.6*10^4^;9.4*10^4^) | 0.048 | 1.39 |
| Methionine | 1.1*10^4^(7.4*10^3^;1.4*10^4^) | 1.4*10^4^(1.2*10^4^;1.8*10^4^) | 0.03 | 1.27 |
| Phenylalanine | 1.7*10^4^(1.3*10^4^;2.4*10^4^) | 2.4*10^4^(1.9*10^4^;2.7*10^4^) | 0.04 | 1.41 |
